# The Role of Gut Microbiota in Tumor Immunotherapy

**DOI:** 10.1155/2021/5061570

**Published:** 2021-08-26

**Authors:** Miao Wu, Jiawei Bai, Chengtai Ma, Jie Wei, Xianjin Du

**Affiliations:** ^1^Department of Emergency, Renmin Hospital of Wuhan University, Wuhan, Hubei, China; ^2^Department of Critical Care Medicine, Renmin Hospital of Wuhan University, Wuhan, Hubei, China

## Abstract

Tumor immunotherapy is the fourth therapy after surgery, chemotherapy, and radiotherapy. It has made great breakthroughs in the treatment of some epithelial tumors and hematological tumors. However, its adverse reactions are common or even more serious, and the response rate in some solid tumors is not satisfactory. With the maturity of genomics and metabolomics technologies, the effect of intestinal microbiota in tumor development and treatment has gradually been recognized. The microbiota may affect tumor immunity by regulating the host immune system and tumor microenvironment. Some bacteria help fight tumors by activating immunity, while some bacteria mediate immunosuppression to help cancer cells escape from the immune system. More and more studies have revealed that the effects and complications of tumor immunotherapy are related to the composition of the gut microbiota. The composition of the intestinal microbiota that is sensitive to treatment or prone to adverse reactions has certain characteristics. These characteristics may be used as biomarkers to predict the prognosis of immunotherapy and may also be developed as “immune potentiators” to assist immunotherapy. Some clinical and preclinical studies have proved that microbial intervention, including microbial transplantation, can improve the sensitivity of immunotherapy or reduce adverse reactions to a certain extent. With the development of gene editing technology and nanotechnology, the design and development of engineered bacteria that contribute to immunotherapy has become a new research hotspot. Based on the relationship between the intestinal microbiota and immunotherapy, the correct mining of microbial information and the development of reasonable and feasible microbial intervention methods are expected to optimize tumor immunotherapy to a large extent and bring new breakthroughs in tumor treatment.

## 1. Introduction

Malignant tumors are one of the major diseases that seriously threaten human health worldwide [1]. The main treatment methods include surgery, radiotherapy, and chemotherapy and targeted therapy. In recent years, with the rapid development of tumor immunity research, immunotherapy has gradually become a promising new anticancer method, mainly represented by programmed cell death-1 (PD-1)/programmed death-ligand 1 (PD-L1) inhibitor [2, 3]. It can achieve better results in the treatment of some advanced tumors, and some patients can even be completely relieved. However, immunotherapy can only be applied to the treatment of a small number of tumors, and a considerable number of patients are not sensitive to this method. In particular, the therapeutic effect of some solid tumors is even more unsatisfactory, and the incidence of immune-related complications is also high. Therefore, how to optimize immunotherapy, improve therapeutic effects, and reduce adverse reactions is the focus of current scientific research. Studies have found that gut microbiota participates in many important physiological activities of the human body, such as digestion, metabolism, defense response, and immune regulation, and plays an eventful role in the process of balancing health and disease, including regulating autoimmunity and malignant tumor progression [4, 5]. The influence of the intestinal microbiota on tumors runs through all stages of occurrence, development, and treatment. The sensitivity and adverse reactions of tumor immunotherapy are closely related to the gut microbiota [6, 7]. This review will focus on the interaction between the gut microbiota and tumor immunotherapy, in order to provide new ideas for optimizing tumor immunotherapy.

## 2. Gut Microbiota and Tumor Immunity

### 2.1. Gut Microbiota

The gut microbiota is an intricate microecology composed of more than 10^14^ microorganisms that coexist with the human body, including bacteria, fungi, and viruses. Because of its close relationship with the human body, it is called the “second genome” of humans [8]. The intestinal microbiota is not static in the human body but will be affected by multiple factors such as diet, drugs, and smoking, and a dynamic balance among various bacterial species can be achieved [9]. With the advancement of genomics and metabolomics technology, the research on the gut microbiome has gradually deepened [6]. The interaction between the intestinal microbiota and the human body was revealed, and it was also found that the intestinal microbiota is closely related to the occurrence and evolution of various diseases [10–12]. The intestinal microbiota plays an important role in the human body environment. It can stimulate the body to produce a large number of lymphocytes and lymphatic tissues, thereby promoting the normal development and gradual maturity of the systemic immune system and mucosal immune system. The imbalance of the intestinal microbiota can promote the development of various malignant tumors [13], such as gastrointestinal malignancies. Most of the intestinal microbes are bacteria, and they can be roughly divided into three categories: beneficial bacteria, neutral bacteria, and harmful bacteria. The beneficial bacteria in the intestines are mainly obligate anaerobic bacteria, *lactobacilli*, *bifidobacteria*, etc. [7]. Among them, obligate anaerobic bacteria accounted for more than 99% of the dominant microbiota in the intestine, mainly including *spirillum*, *peptostreptococcus*, and *Bacteroides*. *Lactobacillus* and *bifidobacteria* are common probiotics, which have been proven to improve the intestinal environment and have a good effect on metabolism, immunity, and neural response [14]. Neutral bacteria are conditional pathogenic bacteria, mainly facultative aerobic bacteria. Facultative aerobes are nondominant intestinal microbiota, such as *Enterococcus* and *Enterobacter*. They are innocuous when the gut microecological balance is normal, but they are aggressive under certain conditions. Harmful bacteria, namely intestinal pathogens, mainly include *Vibrio cholerae*, *Salmonella*, *Shigella*, *Proteus*, and *pathogenic Escherichia coli*. If there are too many harmful bacteria in the human intestinal tract, the immune system will be weakened and even harmful substances such as carcinogens will be produced.

The intestinal microbiota is closely related to the occurrence and development of tumors, and its main mechanism may include releasing toxins to destroy the DNA of normal cells, causing gene mutations and directly leading to cell cancer [15, 16]. For example, the *E. coli* PKS genome encodes the colibactin protein, and the toxin produced by the fragile enterotoxin is related to acute inflammatory bowel disease and colorectal tumors. Similarly, cytotoxic distention toxin (CDT) and colibactin produced by several gram-negative bacteria can cause DNA damage to mammalian cells. In addition, the metabolites produced by microorganisms that promote local chronic inflammation can destroy local cell tissues and induce immune disorders. For example, it is found in liver cancer that lipopolysaccharide (LPS) produced by the intestinal microbiota can activate toll-like receptor 4 (TLR4) to help patients with chronic liver disease progress to tumors. The disorders of the gut microbiota can also affect the expression of major mucin (MUCIN2) on goblet cells. Goblet cells play a key role in intestinal homeostasis. Its destruction is closely related to the occurrence of colorectal cancer [17]. The intestinal microbes can also activate the NF-*κ*B signaling pathway in a variety of ways, leading to an increase in the secretion of many cytokines, such as TNF, IL-1, and IL-6 [18]. The combination of the abovementioned cytokines and their receptors activates the NF-*κ*B pathway. The activation of NF-*κ*B in tumor cells enhances antiapoptotic genes and promotes the survival and proliferation of tumor cells. A variety of microorganisms have been found to be related to gastrointestinal malignant tumors, including *Helicobacter pylori*, *Epstein-Barr virus*, *human papillomavirus*, *Mycoplasma species*, *Escherichia coli*, and *Streptococcus bovis* [9].

### 2.2. Tumor Immunity

Normally, the immune system can distinguish and extirpate tumor cells in the tumor microenvironment (TME). The body's antitumor immune response is cellular immunity and humoral immunity. Helper T cells (Th cells) are the core of immune regulation. Th cells are mainly divided into Th1 cells and Th2 cells. Th1 is involved in cellular immunity, and Th2 is involved in humoral immune response. Among them, cellular immunity is the most important way of immunity. T cells, macrophages, and natural killer (NK) cells are the most important immune cells. In terms of tumor treatment, immunotherapy has achieved shocking clinical success. However, when more patients receive the same treatment, the clinical efficacy is minimal or no effect. The reason is that in the tumor microenvironment on which tumor cells depend for survival, the positive immune function is inhibited, so that normal immune cells cannot attack tumor cells, and tumor immune escape occurs [19].

**Figure 1:**
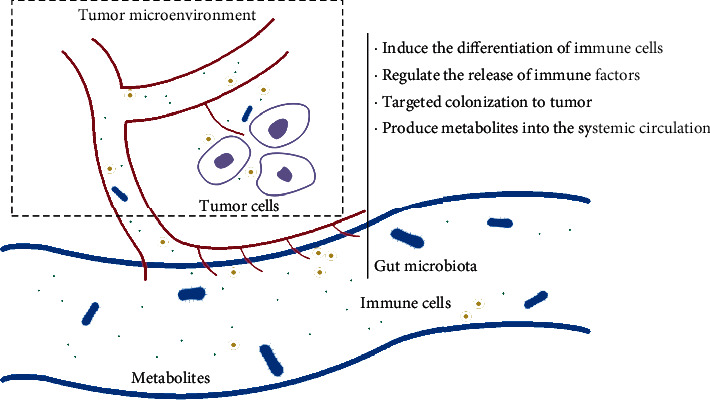
The role of gut microbiota on tumor immunity.

The gut microbiota can influence the occurrence, progress, and prognosis of tumors by regulating the immune balance of the body and the “tumor organismal environment (TOE).” The concept of TOE is derived from the tumor microenvironment. It not only includes tumor cells, immune cells, fibroblasts, intratumoral microorganisms, and cellular metabolites in the local lesion, but also includes systemic immunity, circulation, metabolism, and intestinal microbiota closely related to tumor development [20] (Figure 1). Mutated cells can affect the normal proliferation and differentiation of immune cells (such as CD8^+^ cells, Treg cells, and Th cells) by hiding new antigens, expressing immunosuppressive factors (such as PD-L1, CD80, and CD86), and inducing immune cell dysfunction. This makes TME in an immunosuppressive state, which is an important factor in tumor formation and proliferation [21].

### 2.3. The Influence of Gut Microbiota on Tumor Immunity

Gut microbiota plays an important role in the occurrence and progress of tumors, and the immune system is also the dominant force in tumor control [22]. Studies have shown that the gut microbiota can regulate immune function to play an antitumor effect [23–29]. At present, studies have found that the gut microbiota is related to antitumor immune factors. *Bacteroidetes*, *Akkermansia*, and *Lactobacillus* are positively correlated with antitumor immune factors. In contrast, *Firmicutes*, *Proteobacteria*, and *Parabacteroides* have opposite correlations [30]. A study found that prebiotics can induce antitumor immune responses in mice with melanoma and inhibit tumor growth, while tumor growth in germ-free mice is not affected [31]. This just reflects the important role of intestinal microbes in the antitumor immune response. A study on colon cancer has found that intestinal microbes can stimulate the expression of IL-6 and IL-1*β*, promote the expansion of Th17 cells, and thus increase the resistance to colitis and colon cancer. Even a single bacterial strain, *Odoribacter splanchnicus*, can also exert antitumor immunity [32]. *Lactobacillus HDB1258* isolated from the feces of breastfed infants can play an antitumor effect by activating innate immunity to enhance the immune response, including significantly increasing the cytotoxicity of NK cells and the phagocytosis of macrophages, as well as increasing TNF-*α* and IL-10 expression [33]. In addition, the intestinal microbiota can also regulate the level of chemokines and affect the penetration of CD8^+^ T cells, affecting the survival of patients with melanoma [34]. Supplementing *Bifidobacterium* Strain-Specific can enhance lymphocyte-mediated anticancer immunity to induce anticancer effects [16]. The metabolites of the gut microbiota can also have antitumor immunity activity. For example, short-chain fatty acids (SCFAs) and indole derivatives have shown strong immune and antitumor activity, directly manifested in increasing lymphocytes in peripheral blood, including CD4^+^ and CD8^+^ T cells or NK and NKT cells [35]. The tryptophan metabolites of the gut microbiota can profoundly regulate the host's immune system through the aryl hydrocarbon receptor (AHR), a key regulator of innate and adaptive immune responses, thereby affecting the immune response to tumors [36]. Butyrate is also an intestinal microbial metabolite, which can directly enhance the antitumor cytotoxic CD8 T cell response in vitro and in vivo by modulating the ID2-dependent manner of the IL-12 signaling pathway [37]. The gut microbiota can also modify bile acids, and recent evidence shows that bile acids promote antitumor immune responses by activating and recruiting antitumor immune cells such as natural killer T cells. This indicates that gut microbes can also form antitumor immunity by modifying metabolites [38]. In addition to metabolites, the intestinal microbiota can also target hepatic sinusoidal endothelial cells (LSECs) to regulate the immune tolerance induced by them to prevent liver metastasis of cancer [39].

However, when the internal and external environment of the body changes, the homeostasis of the intestinal microbiota will be destroyed, causing imbalance of the intestinal microbiota. The imbalanced intestinal microbiota will inhibit the immune system to promote the occurrence and development of tumors [40–42]. Microbial disorders can promote chronic inflammation and early T cell failure by overstimulating CD8 T cells, thereby reducing antitumor immunity, resulting in colon tumor susceptibility [43]. After the gastric mucosa is infected with *Helicobacter pylori*, it can cause expression of gastric epithelial cells to promote inflammatory and antimicrobial factors. This defense of gastric epithelial cells can further stimulate the innate immune response from inflammatory reactions and ultimately produce adaptive immune responses. The severity of these reactions is closely related to gastric cancer [44]. Multiple myeloma is a malignant tumor of plasma cells, while the impact of immunomodulatory factors on bone marrow microenvironment may play a role in it. More and more evidence suggested that intestinal microorganisms had an impact on their host adaptability and innate immune system, inflammatory pathway, and bone marrow microenvironment. Therefore, intestinal microbial disorders may affect the occurrence of multiple myeloma [45]. Patients with non-alcoholic fatty liver disease (NAFLD) related cirrhosis are prone to intestinal microbiota disorders. These disordered microorganisms can produce short-chain fatty acids and trigger T cell immunosuppressive phenotypes, which are characterized by regulatory T cell expansion, CD8^+^ T cell attenuation. Disturbance of the intestinal microbiota can induce the occurrence and development of hepatocellular carcinoma (HCC) [46]. In the context of benign liver disease or colitis, the gut microbiome can promote the accumulation of CXCR2 polymorphonuclear myeloid-derived suppressor cells (PMN-MDSCs) in the liver and then control hepatocytes to form an immunosuppressive environment and induce the expression of CXCL1 to promote liver cancer [47].

## 3. Intestinal Microbiota and Tumor Immunotherapy

### 3.1. Tumor Immunotherapy

Tumor immunotherapy includes checkpoint inhibitors (CPIs), lymphocyte-promoting factors, and T cells (such as chimeric antigen receptor T cells), as well as cancer vaccines, oncolytic viruses, and bispecific antibodies. Due to the unique immune escape mechanism of tumors, the immune microenvironment of tumors is often in an immunosuppressive state, that is, most tumors are “cold tumors,” and the overall immune state of the body has not changed much. Therefore, compared with immune enhancement therapy, immune checkpoint inhibitors (ICIs) are obviously more targeted. The representative drugs of CPI are cytotoxic T lymphocyte-associated antigen 4 (CTLA-4) antibody and PD-1/PD-L1 antibody [48]. CTLA-4 antibody can competitively block the binding of CD28 and CD80/86 ligands, thereby interfering with T cell receptor signals and affecting early T cell activation and proliferation and ultimately exerting a tumor suppressor effect. PD-1 is expressed on activated T cells, B lymphocytes, and natural killer cells. It will be phosphorylated after binding to the B7 ligand PD-L1, thereby inhibiting T cell proliferation and related immune responses; thus, targeting PD-1/PD-L1 inhibitor enhances the antitumor immune activity mediated by T cells and ultimately exerts an antitumor effect [49]. The results of clinical trials show that CPI can effectively improve the prognosis of various malignant tumors such as melanoma, lung cancer, gastric cancer, esophageal cancer, and kidney cancer. A review in 2021 compared the efficacy and safety of first-line immune checkpoint inhibitors with platinum-based chemotherapy (with or without bevacizumab) in patients with advanced non-small-cell lung cancer. The review included a total of 15 clinical trials, and the results showed that ICI monotherapy or combination therapy may lead to a higher overall survival rate, but their incidence of adverse reactions is also higher [50]. A multicenter open-label parallel-arm phase II trial (MIRACULUM) evaluated the efficacy and safety of an anti-PD-1 monoclonal antibody, prolgolimab, for patients with advanced melanoma. The result is that prolgolimab shows significant antitumor activity and controllable safety in patients with advanced melanoma [51]. Although the efficacy and safety of CPI have been confirmed, only a small number of patients can benefit from it. The current methods for predicting the effect of immunotherapy are mainly to judge through gene sequencing and pathological examination, such as microsatellite status and tumor mutation burden. However, these methods are still not good at screening people who can benefit from immunotherapy. The difference in intestinal microbiome has been shown to be related to the efficacy of immunotherapy in some studies, making it possible to become a new target for predicting the sensitivity of immunotherapy.

### 3.2. Gut Microbiota Affects the Sensitivity of Tumor Immunotherapy

The influence of the gut microbiota on immune system makes it a pivotal part of the tumor organismal environment, which largely affects the sensitivity of tumors to various treatments, especially immunotherapy [24, 52–57]. The composition of intestinal microbiome has a significant impact on the efficacy of anticancer immune surveillance, which contributes to the therapeutic activity of CTLA-4 or PD-1/PD-L1-based cancer immunotherapy. A systematic review analyzed the impact of the intestinal microbiota on the therapeutic effects of ICIs in a variety of solid tumors [2]. The results showed that patients rich in *Firmicutes* and *Verrucomicrobia* nearly generally had higher sensitivity to ICIs, while patients rich in *Proteobacteria* generally showed unfavorable results. Bacteroidetes and treatment response are mixed correlations. Another study analyzed the feces of patients with advanced non-small-cell lung cancer who received nivolumab in the clinical trials CheckMate-078 and CheckMate-870, which showed there was a significant positive correlation between intestinal microbiota diversity and progression free survival (PFS). *Bifidobacterium longum*, *Prevotella enterica*, and *Alistipes putredinis* were the dominant intestinal strains in patients with treatment sensitivity. It was speculated that the intestinal microbiota enhanced the effect of immunotherapy by enhancing host memory T cells and natural killer cell signals [58]. The identification and functional research of these “beneficial bacteria” may be beneficial to the development of immune synergists, which are used as auxiliary intervention measures for tumor treatment [14]. For example, supplementation of *Bifidobacterium strains* can be used as a strategy to improve the effectiveness of PD-1 inhibitors in the treatment of CRC [16]. There is also a clinical trial that evaluated the safety and efficacy of responder-derived fecal microbiota transplantation (FMT) together with anti-PD-1 in PD-1-refractory melanoma patients, and the results showed that 6 of 15 patients obtained clinical benefits. Respondents showed increased microbial abundance, which was previously shown to be related to the response to anti-PD-1, increased CD8 T cell activation, and decreased frequency of interleukin-8-expressing myeloid cells. By adjusting the intestinal microbiome, the tumor microenvironment is reprogrammed, and the resistance of PD-1 advanced melanoma to anti-PD-1 is overcome [59]. It is worth noting that the mechanism by which the gut microbiota affects tumor immunotherapy is still unclear. Fessler et al. systematically reviewed basic research related to intestinal microbiota and immunotherapy and believed that possible ways for intestinal microbiota to promote the efficacy of immunotherapy include the promotion of tumor-associated antigen recognition, epigenetic regulation of immune cell function, and bystander effect (bacteria-mediated inflammatory stimulation) [60]. *Lactobacillus Johnsonii* and *Olsenella* can significantly improve the efficacy of ICI in four cancer mouse models, which may be related to its metabolite, inosine [61].

### 3.3. Gut Microbiota Affects Adverse Reactions of Tumor Immunotherapy

For tumor immunotherapy with immune checkpoint inhibitors as the main development idea, its treatment method can improve the body's antitumor immunity, but its adverse reactions will involve multiple organ systems such as skin, gastrointestinal tract, pituitary gland, thyroid gland, liver, heart, and lung [62]. The gut microbiota can not only enhance the sensitivity of immunotherapy, but also reduce the adverse effects of these drugs [63–65]. A systematic review has analyzed the effect of the intestinal microbiota on the adverse reactions of ICIs in the treatment of different solid tumors. The study found that *Firmicutes* are associated with a higher incidence of adverse reactions, while *Bacteroidetes* are clearly associated with a lower incidence [2]. The existence of *Bifidobacterium* can reduce the development of colitis caused by ipilimumab therapy. The mechanism may be that the *Bifidobacterium* species can reduce the adverse effects of immunotherapy by inhibiting proinflammatory cytokines [14]. Tanoue et al. isolated 11 rare strains from the feces of healthy people and cocolonized them in the intestinal tract of mice. They found that the above mixed strains could promote the production of CD8^+^ T cells by interferon *γ* through the CD103^+^ dendritic cells and major histocompatibility class IA molecular pathway, thus enhancing the antitumor efficacy of CPI. At the same time, avoid the occurrence of treatment-related enteritis [66]. For patients rich in manifestal and thick walls (group A), it is easier to cause colitis when applying ipilimumab (CTLA-4 inhibitors). Compared to patients with no colitis, Ipilimumab-induced baseline CD4(+) T cell levels are significantly increased, and several inflammatory biomarkers (IL-6, IL-8, and SCD25) are significantly reduced [67]. The above studies have shown that differences in the intestinal microbiota can affect the adverse reactions of immunotherapy. This difference can be a certain type of microbiota or a composition of the microbiota.

## 4. Application of Gut Microbiota in Tumor Immunotherapy

### 4.1. Biomarkers for Predicting the Effect of Tumor Immunotherapy

Some characteristic microbiota can be used as biomarkers to predict the effect of immunotherapy [68–71]. Since the effect of immunotherapy depends on the appropriate intestinal microbiota, the identification of biomarkers which represent the “appropriate” microbiota composition is conducive to the early prediction of immunotherapy effect [72, 73]. A study reviewed clinical trials of the role of the microbiota in the risk, prognosis, and treatment of patients with pancreatic ductal adenocarcinoma (PDAC) and solid tumors. According to the results, microbiome analysis represents a potential trend to enhance antitumor immunity and improve the efficacy of PDAC treatment [74]. Chaput et al. conducted a follow-up study on patients with metastatic melanoma treated with ipilimumab (a CTLA-4 inhibitor) and found that patients with a predominant phylum *Firmicutes* in the gut microbiota have a better treatment effect. The researchers identified 4 representative strains: *Faecalibaterim*, *Gemmiger*, *Clostridium XI Va*, and *Bacteroides*. They can be used as biomarkers to establish models that can predict the efficacy of ipilimumab to a certain extent, and the area under the receiver operating curve (AUROC) reached 0.895 [67]. Studies have also reported that colitis caused by ICI treatment is related to the fecal microbiota metabolism pathway. The polyamine transport pathway and the synthesis pathway of vitamin B1, B2, and B5 are used as biomarkers to predict the incidence of colitis after immunotherapy. It can reach a sensitivity of 70% and a specificity of greater than 80% [75]. Using microbiota characteristics to predict the possible efficacy and adverse reactions of ICI treatment will help the selection of clinical programs and the prevention of adverse events to a certain extent.

### 4.2. Interventions to Improve the Effect of Tumor Immunotherapy

By intervening in the intestinal microecology, the outcome of tumor immunotherapy can be improved. The main clinical methods used for microecological intervention are the rational use of antibiotics, probiotics, prebiotics, and fecal microbiota transplantation (FMT) [76–78]. Most studies have shown that the use of antibiotics is negatively correlated with the clinical response of ICI, especially in the 1-2 months before the start of ICI. There is a significant correlation between the plant-based diet and the enrichment of the “ICI-favoring” gut microbiome [2]. MSI negative CRC is relatively resistant to immunogenic cell death mediated by immune checkpoint inhibitors. Fidelle et al. used cytotoxicants to adjust the ileal microbiome to immunogenic bacteria. This manipulation leads to a conversation between productive Tfh and B cells in the mesenteric lymph nodes, which ultimately leads to a tumor-specific memory CD8^+^ T cell response and restores sensitivity to immune checkpoint inhibitors [79]. It was observed in the mouse tumor model that a gel made of inulin can regulate the intestinal microbial group, induce systemic memory T cell responses and amplify the antitumor activity of the checkpoint inhibitor antiprogrammed cell death protein-1 (*α*-PD-1). The relative abundance of key symbiotic microorganisms and its short-chain fatty acid metabolites were added by orally inulin-gel [80]. Traditional Chinese medicine has been used to prevent and treat diseases in China for thousands of years. The intestinal microbiota has become a new way to understand Chinese medicine. In various cancers, Chinese medicine can exert anticancer effects by affecting the intestinal microbiota [81–83]. In a mouse colorectal cancer model, Sini Decoction (SND), a classic prescription of traditional Chinese medicine, can upregulate the expression of CD8 T lymphocytes in the colonic mucosa, inhibit the expression of CD4 T cells and inflammatory cytokines in CRC tissue, and then effectively intervene in the development of CRC. And this may be related to its ability to change the abundance of the mouse intestinal microbes. It can reduce the abundance of *Bacteroides fragilis* and *Sulphate-reducing bacteria* and increase the abundance of *Lactobacillus*, *Bacillus coagulans*, *Akkermansia muciniphila*, and *Bifidobacterium* [84].

Some studies have shown that supplementation of “beneficial bacteria” or FMT can increase the sensitivity of tumor immunotherapy, and that “beneficial bacteria” or suitable fecal microbiota can be made into medicaments, which are expected to be used in clinical adjuvant therapy. Tanoue et al. isolated eleven strains of bacteria from healthy human feces and then used them in mice to induce CD8^+^ T cells that can secrete IFN-*γ* and enhance the effect of ICI treatment [66]. The results are currently in the clinical transformation test stage. FMT is a more thorough microbiota intervention method, which can reshape the gut microbiota of patients. Davar et al. found that FMT derived from responders and anti-PD-1 together can regulate the intestinal microbes and reprogram the TME, so that patients with PD-1-refractory melanoma can obtain clinical benefits [59]. Another team transplanted the fecal microbiota of sensitive patients to patients with malignant melanoma who were not sensitive to PD-1 inhibitors and achieved good clinical effects after immunotherapy again. Based on the relationship between immunotherapy and intestinal microbiota, formulating personalized immunotherapy programs for patients based on the characteristics of intestinal microbiota may be a way to optimize tumor treatment [85]. More clinical studies are ongoing. More than 10 items have been registered on the clinicaltrials.gov website, as shown in Table 1.

### 4.3. Drug Carriers for Enhancing the Effect of Tumor Immunotherapy

In recent years, nanotechnology and gene editing technology have gradually matured, and certain strains can be used as drug carriers to enhance the antitumor effect of drugs [6]. Some studies load genes expressing PD-1 antibody and CTLA-4 antibody into *Salmonella*, which can improve the efficiency of drug delivery, realize the combined use of multiple immunotherapies, and improve the efficacy [86]. A team has packaged anti-CD47 nanoantibodies in engineered nonpathogenic *Escherichia coli strains*, which can specifically release antibodies after being lysed in the tumor microenvironment. In mouse lymphoma models, it can enhance tumor infiltrating T cell activation, inhibit tumor growth and metastasis, and prolong the survival time of mice [87]. Due to the ability of bacteria to move and proliferate, using bacteria as a drug delivery carrier can better achieve targeted drug delivery and sustained drug release. The apposite use of the interaction between bacteria, the immune system, and tumor cells may greatly enhance the effectiveness of immunotherapy. However, this technology has certain risks, such as the possibility of bacterial infections, uncontrollable proliferation, hidden biological safety hazards, and the mutual influence of bacterial immunity and tumor immunity, and so on.

## 5. Conclusion

Immunotherapy has made great breakthroughs in the treatment of some epithelial tumors and hematological tumors. However, its adverse reactions are common or even more serious, and the reaction rate in some solid tumors is not ideal. With the maturity of genomics and metabolomics technologies, the role of intestinal microbiota in tumor development and treatment has gradually been recognized. The microbiota may affect tumor immunity by regulating the host immune system and tumor microenvironment. The effect and complications of tumor immunotherapy are related to the composition of the intestinal microbiota. The composition of intestinal microbiota that is sensitive to treatment or prone to adverse reactions has certain characteristics. They can be used as biomarkers to predict the prognosis of immunotherapy and can also be used as “immune enhancers” to assist immunotherapy. Microbial intervention, including microbial transplantation, can improve the sensitivity of immunotherapy or reduce adverse reactions to a certain extent. In recent years, there have been more and more researches related to the design and development of engineered bacteria that contribute to immunotherapy. Based on the relationship between the intestinal microbiota and immunotherapy, the correct mining of microbial information and the development of reasonable and feasible microbial intervention methods are expected to optimize tumor immunotherapy to a large extent and bring new breakthroughs in tumor treatment.

## Figures and Tables

**Table 1:** Clinical research on intestinal microbiota and tumor immunotherapy.

Study type	NCT number	Condition or disease	Immunotherapeutic drugs	Microbiological interventions	Purpose	Estimated enrollment	Primary outcome	Study design	Location
Interventional	NCT04163289	Renal cell carcinoma	Ipilimumab/nivolumab	FMT	To evaluate the efficacy and safety of FMT in the prevention of adverse reactions in immunotherapy	20	Occurrence of immune-related colitis associated with ipilimumab/nivolumab treatment	Single group Open label	Canada
	NCT04130763	Gastrointestinal system cancer	PD-1 inhibitor	FMT	To determine whether the FMT capsule improves the response rate of anti-PD-1 treatment	10	Objective response rate Rate of abnormal vital signs and laboratory test results The number of adverse events	Single group Open label	China
	NCT03353402	Melanoma stage IV Unresectable stage III melanoma	Unspecified	FMT	Altering the gut microbiota of melanoma patients who failed immunotherapy using FMT from responding patients	40	Incidence of FMT-related adverse events Proper implant engraftment	Single group Open label	Israel
	NCT04264975	Solid carcinoma	Unspecified	FMT	Part 1: development of microbiome biomarkers for immuno-oncology; part 2: proof-of-concept trial on the fecal microbiota transplantation in patients who are being treated with immunotherapy for advanced solid tumor	60	Overall response rate	Single group Open label	Korea
	NCT03341143	Melanoma	Pembrolizumab	FMT	To determine if the FMT improves the body's ability to fight your cancer	20	Objective response rate	Single group Open label	United States
	NCT03829111	Advanced renal cell carcinoma Clear cell renal cell carcinoma Metastatic renal cell carcinoma Stage III renal cell cancer AJCC v8 Stage IV renal cell cancer AJCC v8 Unresectable renal cell carcinoma	Nivolumab Ipilimumab	Clostridium butyricum CBM 588 probiotic strain	To determine the effect of clostridium butyricum CBM 588 probiotic strain (CBM 588) (in combination with nivolumab/ipilimumab) on the gut microbiome in patients with metastatic renal cell carcinoma (mRCC)	30	Change in Bifidobacterium composition of stool	Randomized Parallel assignment Open label	United States
	NCT03686202	All solid tumors	PD-1/PD-L1 inhibitor	MET-4	To assess the safety, tolerability and engraftment of MET-4 strains when given in combination with immune checkpoint inhibitors	65	Cumulative relative abundance of immunotherapy-responsiveness associated species Changes in relative abundance of immunotherapy-responsiveness associated MET-4 strains Number of participants with treatment-related adverse events	Randomized Single group Open label	Canada
Observational	NCT04136470	Non-small-cell lung cancer Microbiome Metagenome Immunotherapy Melanoma	Nivolumab Ipilimumab Atezolizumab	/	To develop and validate the BioForte technology. Its main functionality should be to in silico determine candidates for novel microbiome-based therapeutics and diagnostics.	130	Microbial diversity in stool samples Number of responders and nonresponders on immunotherapy	Case-only Cross-sectional	Poland
	NCT03797170	Diffuse large B cell lymphoma	Front-line R-CHOP (rituximab-cyclophosphamide, doxorubicin, vincristine, prednisone)	/	To study the functional gut microbiota layout in association with specific patterns of treatment response in de novo DLBCL undergoing standard first line chemoimmunotherapy	50	Gut microbiota dysbiosis assessment (bacterial DNA of gut microbiota in all patients)	Cohort Prospective	Italy
	NCT03557749	Immune and microbial reconstitution Systemic viral infection Acute-graft-versus-host disease Chronic graft-versus-host-disease Recurrent malignancy Cytokine release syndrome Allogenic related donors Cell therapy/immunotherapy patients	Hematopoietic cell transplant Cell therapy Novel immunotherapy	/	To monitor the immune and microbial reconstitution in hematopoietic cell transplantation and novel immunotherapies	1600	Immune function after hematopoietic cell transplant Immune function after cell therapy/immunotherapy Correlate immune parameters Correlate microbiota changes and their interactions with the host with outcomes of hematopoietic cell transplant	Cohort Prospective	United States
	NCT04169867	Melanoma Healthy volunteers Microbiome Metagenome Immunotherapy	Nivolumab Ipilimumab Atezolizumab	/	To observe the gut microbiota and diet of melanoma patients receiving immunotherapy	1160	Microbial diversity in stool samples Eating habits and health survey	Case-only Cross-sectional	Poland

FMT: fecal microbiota transplantation; /: none.
